# Quality of life, mental health and health beliefs in haemodialysis and peritoneal dialysis patients: Investigating differences in early and later years of current treatment

**DOI:** 10.1186/1471-2369-9-14

**Published:** 2008-11-14

**Authors:** M Ginieri-Coccossis, P Theofilou, C Synodinou, V Tomaras, C Soldatos

**Affiliations:** 1 1st Department of Psychiatry, Medical School, University of Athens, Athens, Greece; 2 Department of Psychology, Panteion University, Athens, Greece; 3 Mental Health Care Unit, Evgenidion Hospital, University of Athens, Athens, Greece

## Abstract

**Background:**

The study examines differences regarding quality of life (QoL), mental health and illness beliefs between in-centre haemodialysis (HD) and continuous ambulatory peritoneal dialysis (CAPD/PD) patients. Differences are examined between patients who recently commenced treatment compared to patients on long term treatment.

**Methods:**

144 End-Stage Renal Disease (ESRD) patients were recruited from three treatment units, of which 135 provided full data on the variables studied. Patients consisted of: a) 77 in-centre haemodialysis (HD) and 58 continuous ambulatory peritoneal dialysis (CAPD/PD) patients, all currently being treated by dialysis for varied length of time. Patients were compared for differences after being grouped into those who recently commenced treatment (< 4 years) and those on long term treatment (> 4 years). Next, cases were selected as to form two equivalent groups of HD and CAPD/PD patients in terms of length of treatment and sociodemographic variables. The groups consisted of: a) 41 in-centre haemodialysis (HD) and b) 48 continuous ambulatory peritoneal dialysis (CAPD/PD) patients, fitting the selection criteria of recent commencement of treatment and similar sociodemographic characteristics. Patient-reported assessments included: WHOQOL-BREF, GHQ-28 and the MHLC, which is a health locus of control inventory.

**Results:**

Differences in mean scores were mainly observed in the HD patients with > 4 years of treatment, providing lower mean scores in the QoL domains of *physical health, social relationships *and *environment*, as well as in overall mental health. Differences in CAPD/PD groups, between those in early and those in later years of treatment, were not found to be large and significant. Concerning the analysis on equivalent groups derived from selection of cases, HD patients indicated significantly lower mean scores in the QoL domain of *environment *and higher scores in the GHQ-28 subscales of *anxiety/insomnia *and *severe depression*, indicating more symptoms in these areas of mental health. With regards to illness beliefs, HD patients who recently commenced treatment provided higher mean scores in the dimension of *internal *health locus of control, while CAPD/PD patients on long term treatment indicated higher mean scores in the dimension of *chance*. Regarding differences in health beliefs between equivalent groups of HD and CAPD/PD patients, HD patients focused more on the dimension of *internal *health locus of control.

**Conclusion:**

The results provide evidence that patients in HD treatment modality, particularly those with many years of treatment, were experiencing a more compromised QoL in comparison to CAPD/PD patients.

## Background

Populations facing chronic illness have been reported to have poorer QoL and mental health including higher levels of depression [1-3]. In this context, health services for ill-health populations including End-Stage Renal Disease (ESRD) have drawn attention to quality of life issues and promotion of mental health [4-6]. It is worth noting that ESRD is a disease with serious effects on the patients' QoL, negatively affecting their social, financial and psychological well-being [7,8].

Regarding relevant research, renal patients undergoing HD or CAPD/PD treatment were found to experience QoL deficits, while the areas affected were shown to be varied [9,10]. Patients' health-related QoL in these two treatment modalities was reported as being comparable; however treatment-related differences were indicated in certain QoL domains for each treatment modality respectively. Findings are mixed with some studies showing that HD patients report better physical well-being, sleep and sexual relationships [11,12]. Such results were indicated for the first two years of dialysis and over time [11,13,14]. However, adverse symptoms such as nocturnal distress and inability to sleep during the nights leading up to dialysis have also been reported by HD patients [15-17]. On the other hand, compromised physical well-being in CAPD/PD patients has been indicated in connection to lower levels of albumin and health complications, e.g. peritonitis [13,18,19].

Regarding mental health, HD patients have been found to experience more depressive symptoms than PD [17,20]. Depression may be linked to the HD treatment modality, since the patient has to be continually connected to the haemodialysis machine during dialysis and so experience significant restrictions in independent living [17,20]. In addition, the rate of reported suicide in HD is higher, while a substantial number of deaths resulting from dietary violations could also be accounted for as suicide [21,22].

Furthermore, HD patients are reported to face psychosocial problems, which can contribute to conflictions between themselves and their medical carers. Such findings could be attributed in part to the stressful conditions in the HD treatment modality, including frequent visits and prolonged waiting time in the dialysis unit [17,23,24].

Regarding psychological dimensions in ESRD, it seems that CAPD/PD patients are better adjusted than HD. This may be due to the peritoneal treatment modality offering increased autonomy and control, flexibility in everyday life and the dietary regime, as well as fewer social restrictions [14,25-27]. PD patients have been found to report better QoL ratings in specific areas like 'perceived ability to travel', 'financial concerns', 'restriction in eating and drinking' and 'dialysis access problems' [11]. Furthermore, PD patients have indicated more positive ratings in several disease QoL domains, e.g. less kidney disease burden, and being more encouraged and satisfied with care [28].

It is recognized that regardless of the treatment method, patients suffering from ESRD have to cope with many adversities, e.g. physical symptoms, specific dietary regimes and changes in their body image, while their control over treatment cannot always be predicted [29,30]. Such constraints are expected to affect the patients' life and physical and social functioning, leading patients to reconsider their personal and professional goals within the context of living with a chronic illness. Subjective issues of this kind can be investigated in patient-reported health beliefs and in perceived physical, psychological and social well-being.

Although a considerable number of articles on ESRD have been published, there are a limited number of studies comparing generic domains of QoL and mental health factors, or health beliefs in HD and CAPD/PD patients. In addition, the outcomes reported from QoL studies are frequently inconclusive. The aim of the present study was to investigate a) self-reported physical, psychological, social and environmental well-being, b) mental health factors such as anxiety and depression, as well as c) illness beliefs of health locus of control in haemodialysis and peritoneal dialysis patients. Differences were examined between patients who recently commenced treatment and those on long-term treatment (early and later years of current treatment).

## Method

### The sample

A cohort of 144 end-stage renal disease patients were recruited from dialysis units within three General Hospitals located within the broader area of Athens. From this cohort, 135 patients provided full data on the variables studied, while the remaining 9 patients were excluded having missing/incomplete data, which could be probably explained by advanced age (mean age of 67.16). The cohort of 135 participants that participated in the analysis consisted of: a) 77 patients (57.0%) undergoing in-centre haemodialysis (HD) and b) 58 patients (43.0%) undergoing continuous ambulatory peritoneal dialysis (CAPD/PD), (the term PD will be used for the remainder of the text). Patients within these two treatment modalities had low comorbidity and were undergoing current dialysis for a varied period of time. In this respect, participants could be categorized into four distinct groups regarding current treatment: a) HD patients who recently commenced treatment (< 4 years), b) HD patients on long term treatment (> 4), c) PD patients who recently commenced treatment (< 4 years) and d) PD on long term treatment (> 4). A cut-off period of 4 years of treatment was agreed upon by the researchers because it was considered that a period of 3–4 years is required for patient adjustment to the diagnosis and treatment of a chronic illness. The group of HD patients with greater than 4 years of treatment included participants with a higher mean length of treatment measured in years (12.65 ± 6.64) in comparison to the respective group of PD patients (5.82 ± 1.13). Sociodemographic characteristics of these groups are presented in Table 1.

**Table 1:** Descriptive characteristics of HD and PD patients in early and later years of treatment.

	**HD** **< 4 years of treatment** **N = 39**	**HD** **> 4 years of treatment** **N = 38**	**PD** **< 4 years of treatment** **N = 41**	**PD** **> 4 years of treatment** **N = 17**
**Age (years)**				
21–40	10 (25.6%)	5 (13.1%)	3 (7.3%)	0 (0%)
41–60	14 (35.8%)	9 (23.6%)	13 (31.7%)	6 (35.2%)
> 60	15 (38.4%)	24 (63.1%)	25 (60.9%)	11 (64.7%)
Total	39 (100.0%)	38(100.0%)	41(100.0%)	17(100.0%)
Mean ± SD	52. 95 ± 16.66	60.53 ± 13.59	62.71 ± 13.37	67.06 ± 9.21
				
**Gender**				
Male	29 (74.3%)	22 (57.8%)	22 (53.6%)	7 (41.1%)
Female	10 (25.6%)	16 (42.1%)	19 (46.3%)	10 (58.8%)
Total	39 (100.0%)	38(100.0%)	41(100.0%)	17(100.0%)
				
**Marital status**				
Single	11(28.2%)	8 (21.0%)	5 (12.1%)	1(5.8%)
Married	26 (66.6%)	26 (68.4%)	34 (82.9%)	13 (76.4%)
D/W/R*	2 (5.1%)	4 (10.5%)	2 (4.8%)	3 (17.6%)
Total	39(100.0%)	38(100.0%)	41(100.0%)	17(100.0%)
				
**Education**				
Elementary	17 (43.5%)	21(55.2%)	12(29.2%)	8 (47.0%)
Secondary	17(43.5%)	8 (21.0%)	20 (48.7%)	9 (52.9%)
University	5 (12.8%)	9 (23.6%)	9 (21.9%)	0 (0%)
Total	39(100.0%)	38(100.0%)	41(100.0%)	17(100.0%)
Mean ± SD	8.44 ± 4.15	8.37 ± 5.0	10.61 ± 3.9	7.76 ± 3.5
				
**Length of treatment**				
Mean ± SD	2.10 ± 1.16	12.65 ± 6.64	2.17 ± 1.13%	5.82 ± 1.13

*D/W/R = Divorced/Widowed/Roommate

Next, in order to investigate differences between the HD and PD treatment modalities without the possible effect of length of treatment, 41 cases of HD and 48 cases of PD patients were selected from the total cohort of 135 participants according to specified criteria to formulate two equivalent groups. Selection criteria included the patient commencing dialysis treatment within a 4 year period and ensured a balanced ratio of male/female participants within the two groups. Following the selection procedure, the two groups were tested for significant differences regarding sociodemographic variables. As seen in Table 2, the groups can be considered equivalent with no statistically significant differences between them (p. > 0.05).

**Table 2:** Characteristics of HD and PD patients selected to form equivalent groups.

	**HD** **N = 41 (46.06%)**	**PD** **N = 48 (53.9%)**	**p-value**
**Age (years)**			
Mean ± SD	65.34 ± 8.37	64.10 ± 10.36	NS
			
**Gender**			
Male	21 (51.30%)	23 (47.90%)	NS
Female	20 (48.70%)	25 (52.10%)	
Total	41 (100.0%)	48 (100.0%)	
			
**Marital status**			
Single	4 (9.75%)	6 (12.50%)	NS
Married	33 (80.48%)	38 (79.20%)	
D/W/R*	4 (9.75%)	4 (8.30%)	
Total	41 (100.0%)	48 (100.0%)	
			
**Education**			
0–9 years	29 (70.73%)	26 (54.16%)	NS
> 9 years	12 (29.27%)	22 (45.84%)	
Total	41 (100.0%)	48 (100.0%)	
			
**Length of dialysis treatment **(years)			
Mean ± SD	4.15 ± 2.57	3.62 ± 2.00	NS

*D/W/R = Divorced/Widowed/Roommate

**NS = p **> 0.05

This table displays the results of the Independent-Samples T-Test demonstrating that there are no statistically significant differences between the HD and PD groups, including patients who were selected according to specified criteria of being in the initial stages of treatment and having similar socio-demographic characteristics.

All participants were of Greek nationality who gave signed consent for participation in the study. They had been informed of their rights to refuse or to discontinue participating in the study according to the ethical standards of the Helsinki Declaration (1983) [31]. Ethical permission for the study was obtained from the scientific committees of the participating hospitals.

### Instruments

The following measurement tools were administered to all patients:

1) The *World Health Organization Quality of Life *questionnaire (WHOQOL-BREF) [32]. It is a self-report inventory of generic QoL with 26 original items and 4 additional items derived from the validation of the instrument within Greek populations [33,34]. The items fall into four domains: a) *Physical Health*, b) *Psychological Health*, c) *Social Relationships *and d) *Environment*, while two items provide a measurement of an *Overall QoL/Health *facet. The scale has demonstrated good internal consistency with Cronbach's alpha ranging from 0.67 – 0.81 per domain. It is rated on a 5-point Likert scale and the range of scores is between 1–20 with higher scores indicating better QoL.

2) The *General Health Questionnaire *(GHQ-28) is a widely used self-report measure of general health, developed by Goldberg (1978) [35]. The questionnaire has demonstrated good psychometric properties within Greek populations (internal consistency, validity with indices of sensitivity, specificity, positive predictive value, negative predictive value and overall misclassification rate) [36]. Besides being used as a screening tool for psychiatric cases in medical settings and general practice, it may also be used to identify self-perceived differences in mental health. The 28-item version used in this study consists of four sub-scales: a) *somatic symptoms*, b) *anxiety/insomnia*, c) *social dysfunction and *d) *severe depression*. Lower scores indicate better mental health.

3) The *Multidimensional Health Locus of Control *(MHLC) is a self-report inventory measuring beliefs on health locus of control. It consists of 18 items, grouped into four factors representing four categories of health beliefs: a) *internal health locus*, b) *chance*, c) *doctors *and d) *important others*. The last three refer to external health locus of control [37,38]. The four factors are not mutually exclusive and scores may weight in a particular direction. Higher scores in one factor indicate a stronger presence of beliefs in the specific dimension. The questionnaire has been translated into Greek and is under validation. Preliminary evidence shows that the 4 factor model of *internal, chance, doctors *and *powerful others *may fit within the data with Cronbach's alpha ranging from 0.50 – 0.85 (authors' communication).

### Statistical Analysis

Kolmogorov-Smirnov tests were performed in order to check whether the values of the sample would fall within a normal distribution. Next, the analyses performed aimed to:

a) Investigate differences between patients who had recently commenced treatment and those on long term treatment within the HD and PD modalities. Thus, one-Way ANOVA analysis was performed to investigate differences within patients falling into 4 categories according to treatment modality and length of treatment. Investigation of differences included QoL domains, mental health and illness beliefs with regards to health locus of control. Next, independent Samples T-tests were performed to check differences between patients who recently commenced treatment and those on long term treatment within each treatment modality. Also, ANCOVA analysis was performed controlling for length of treatment in the PD and HD patients.

b) Investigate differences between HD and PD patients, using two groups comprised of selected cases from the total cohort of 135 patients, equivalent for length of treatment and sociodemographic characteristics. Independent Samples T-tests were performed in order to check for significant differences in all variables investigated in the study.

## Results

The values of the total cohort were found to pass the normality distribution test. Regarding differences within the four patient groups consisting of HD and PD patients who recently commenced and those on long term treatment (early/later years of treatment), One-Way ANOVA analysis was conducted. The results of this test reveal that HD patients with more than 4 years of current treatment varied significantly from the other patient groups indicating lower QoL mean scores in the WHOQOL-BREF domains of *physical health, social relationships *and *environment *(Table 3).

**Table 3:** WHOQOL-BREF, GHQ-28 and MHLC in HD and PD patients in early and later years of treatment.

**WHOQOL-BREF domains**	**HD** **< 4 years of treatment(N = 39)** **M ± SD**	**HD** **> 4 years of treatment (N = 38)** **M ± SD**	**PD** **< 4 years of treatment (N = 41)** **M ± SD**	**PD** **> 4 years of treatment(N = 17)** **M ± SD**	**p-value**
Physical	13.86 ± 3.16	11.95 ± 3.76	13.92 ± 2.87	13.07 ± 2.80	0.03*
Psychological	14.34 ± 3.02	12.29 ± 3.96	13.23 ± 3.01	13.64 ± 3.35	NS
Social relationships	13.88 ± 2.86	11.77 ± 4.05	14.13 ± 2.33	13.56 ± 2.54	0.00*
Environment	13.05 ± 2.64	12.65 ± 2.82	14.69 ± 1.76	14.05 ± 1.82	0.00*
Overall QoL/health	3.10 ± 0.97	2.88 ± 1.18	3.16 ± 0.79	3.11 ± 0.91	NS
					
**GHQ – 28 subscales**					
					
Somatic symptoms	1.76 ± 0.52	1.94 ± 0.54	1.66 ± 0.52	1.78 ± 0.54	NS
Anxiety/insomnia	1.95 ± 0.68	1.87 ± 0.62	1.49 ± 0.54	1.49 ± 0.72	0.00*
Social dysfunction	2.22 ± 0.51	2.35 ± 0.43	2.17 ± 0.31	2.20 ± 0.47	NS
Severe depression	1.46 ± 0.64	1.64 ± 0.80	1.28 ± 0.53	1.32 ± 0.63	NS
Total score	1.85 ± 0.45	1.95 ± 0.50	1.65 ± 0.42	1.80 ± 0.48	0.04*
					
**Health Locus of Control factors**					
					
Internal locus	27.82 ± 6.20	25.67 ± 7.33	25.10 ± 8.03	21.46 ± 8.41	0.04*
Chance	25.15 ± 8.33	23.59 ± 7.63	20.94 ± 8.87	28.40 ± 7.90	0.01*
Doctors	16.23 ± 2.42	15.43 ± 2.84	17.25 ± 1.35	16.26 ± 2.08	0.00*
Important others	12.38 ± 4.21	11.64 ± 4.43	12.51 ± 4.31	11.06 ± 5.76	NS

*p < 0.05; N = 135.

This table displays the results of One-Way ANOVA demonstrating differences between HD and PD patients classified into the categories of patients who recently commenced treatment and patients on long term treatment.

In addition, according to the means of the GHQ-28 subscales, the group of HD patients with less than 4 years of treatment indicated the highest level of *anxiety *and *insomnia*, while those with more than 4 years of treatment indicated the highest mean in the GHQ-28 total score, signifying poorer overall mental health (Table 3).

A series of independent samples T-tests were then performed between the groups of patients who recently commenced and those in long term treatment and for each modality separately. Significant differences were found between the groups of the patients in the HD treatment modality. Specifically, HD patients on long term treatment had significantly lower scores in the domains of *physical, psychological and social well-being*. On the other hand, no significant differences in QoL and mental health parameters were found between the two PD groups of patients. It should be noted that differences in length of treatment between the PD groups were not as great as between the HD groups (approximately 3.5 years and 10 years respectively) as mentioned before and seen in Table 1.

Concerning illness beliefs with regards to health locus of control, the group of HD patients who had recently commenced treatment differed significantly from patients in the other categories, indicating the highest mean scores in the dimension of *internal *heath locus of control (mean scores and p-values can be seen in Table 3). As for the groups of PD patients, those with more than 4 years of treatment indicated the highest mean score in the dimension of *chance*. Concerning the dimension of d*octors*, significant differences among the four groups were indicated. So, it is observed that HD patients with more than 4 years of treatment have the lowest mean score, while PD patients who had recently commenced treatment have the highest mean score in this dimension.

In order to test for the effect of length of treatment, ANCOVA analysis was applied controlling for years of treatment in the total cohort. The effect was observed in the WHOQOL-BREF domain of *environment*, in the GHQ-28 subscales of *anxiety/insomnia *and *severe depression *and in the dimensions of *internal *and *doctors *regarding health locus of control.

The analysis conducted up to this point reveals that the variable of length of treatment plays a significant role in the patients' quality of life. Consequently, differences between HD and PD patients were further examined using the selective criteria of less than 4 years of treatment combined with similar sociodemographic characteristics. Based on selection of cases from the total cohort, two equivalent groups were formed comprising of PD and HD patients and were tested for significant differences. Independent Samples T-test provided evidence of no significant differences in gender, age, marital status, education and length of treatment. Table 2 shows the characteristics of these groups.

Following investigation into QoL and mental health, independent samples T-tests were then performed between the two groups of patients showing statistically significant differences. Specifically, HD patients indicated significantly lower QoL scores in the *environment *domain of WHOQOL-BREF. Furthermore, they reported significantly higher scores in the GHQ-28 sub-scales of *anxiety/insomnia*, *severe depression *and the total GHQ-28 score (See Table 4).

**Table 4:** WHOQOL-BREF, GHQ-28 and MHLC in HD and PD equivalent groups.

**WHOQOL-BREF domains**	**HD patients (N = 41)** **M ± SD**	**PD patients (N = 48)** **M ± SD**	**p-value**
Physical	12.44 ± 3.78	13.46 ± 2.78	NS
Psychological	12.91 ± 3.51	13.16 ± 3.16	NS
Social relationships	13.06 ± 2.89	13.69 ± 2.21	NS
Environment	12.95 ± 2.63	14.35 ± 1.83	0.00*
Overall QoL/health	2.98 ± 0.98	3.11 ± 0.84	NS
**GHQ-28 subscales**			
			
Somatic symptoms	1.86 ± 0.58	1.73 ± 0.55	NS
Anxiety/insomnia	1.87 ± 0.69	1.53 ± 0.64	0.02*
Social dysfunction	2.36 ± 0.55	2.22 ± 0.36	NS
Severe depression	1.65 ± 0.88	1.32 ± 0.60	0.04*
Total score	1.93 ± 0.57	1.70 ± 0.48	0.04*
**Health Locus of Control factors**			
			
Internal locus	27.36 ± 7.00	23.15 ± 8.35	0.01*
Chance	25.21 ± 8.65	23.22 ± 9.16	NS
Doctors	16.48 ± 2.27	16.80 ± 1.72	NS
Important others	13.21 ± 4.56	11.80 ± 4.77	NS

*p < 0.05; N = 89.

This table displays the results of the Independent-Samples T-Test demonstrating differences between HD and PD patients groups, both in initial stages of treatment and with similar socio-demographic characteristics.

With regards to illness beliefs, a significant difference was observed with HD patients presenting higher scores in the dimension of *internal *health locus of control. Both groups presented a similar pattern of illness beliefs, according to which higher values were identified in the *internal *and *chance *dimensions followed by the dimensions of *doctors *and *important others *(See Table 4).

## Discussion

Deterioration in quality of life is particularly evident in the HD group of patients who have been on dialysis for an extended period of time. Most quality of life domains seem to be affected, including overall mental health. After taking into consideration that QoL deficits were mostly indicated by HD patients who had been on long term treatment, it could be argued that these patients experienced significant QoL changes over time, including deterioration in *physical, social and environmental well-being *as well as in overall mental health. In the relevant literature, it has been suggested that health-related QoL was more compromised in HD patients, which may only become evident as time on therapy increases [39].

In particular, HD patients seem to experience a higher level of adverse symptoms such as *anxiety *and *insomnia *during the initial years of treatment, which may reflect the patient's emotional burden in having to adhere to a strict treatment regimen. It is suggested that longitudinal studies using samples that can be available over time, may be useful for overcoming cross-sectional limitations and acquiring robust evidence on QoL differences between HD and PD treatment modalities over a period of time. This is an issue of increased interest as there is evidence for changes in QoL, including a more favourable effect of HD treatment on the patients' physical well being over the first year [11]. However, it not known whether this improvement is maintained.

Regarding the PD groups, differences in QoL and mental health between patients who recently commenced and those on long term treatment are not significant. However, one should take into consideration that the PD groups of this sample did not have a large difference in terms of length of treatment, as was the case with the HD groups. It is argued that quality of life differences linked to modality without taking into consideration the time factor may restrict our understanding of differences and changes in QoL over time.

Following this line of thought, QoL controlling for the effects of length of treatment was investigated (ANCOVA). Accordingly, it was observed that patients' quality of life was only affected in the *environment *domain, and in the *anxiety/insomnia *dimension of mental health. Focusing further on the differences between HD and PD patient groups, both in initial stages of treatment, it became evident that QoL deficits in these two treatment modalities were more limited.

Accordingly, HD patients reported a more compromised quality of life indicating a higher dissatisfaction with different aspects of their *environment*. Specifically, these patients reported experiencing various difficulties, including a more negative opinion about availability and quality of *health services*. Also, they reported increased dissatisfaction with their *finances, opportunities for recreation, means of transportation*, as well as *opportunities for acquiring everyday living information and new skills*. Deficits in this area of QoL may be associated with treatment, since in-center haemodialysis imposes serious restrictions on the patient's life due to dependence on the dialysis machine. Also, dissatisfaction with health services may reflect the burden experienced by these patients, including stressful conditions during the dialysis procedure, high frequency of received in-center treatment and prolonged waiting-time in the unit [17,23,24]. It is noted that the WHOQOL-BREF domain of *environment *and the facets mentioned above seem to accurately represent the dimensions, which may be seriously affected by dialysis treatment procedures.

These results correspond to previous findings showing that it is the PD patients who report better ratings in 'perceived ability to travel', 'financial concerns', and 'dialysis access' [11], indicating increased satisfaction with their health care [28].

Furthermore, focusing on differences between HD and PD equivalent groups of patients in initial stages of treatment, HD patients reported more symptoms of *anxiety, sleeping problems, depression *or *suicidal ideation*. Problems or patterns of sleep are a central issue for many disorders and they are frequently addressed for ill as well as healthy populations [11,40]. Regarding the comparatively higher level of suicidal thoughts reported by this category of patients, this is a finding in agreement with international literature indicating higher rates of suicidal attempts in HD patients [21,22]. However, findings in the relevant literature are mixed regarding physical and psychological well-being and certain studies have suggested that HD patients reported a more positive evaluation of their physical well-being [12].

Regarding health beliefs, HD patients indicated a greater preference for the *internal *dimension focusing more on their own personal control to regulate their health condition (Table 4). This may reflect a stronger need of these patients to counterbalance the imposed dependence on the dialysis procedure and the restrictive dietary regimen by exercising control over their illness. Also, it could be the case that beliefs focusing on personal control and responsibility over one's condition could indicate self-blame and depressive mood. HD patients are reported in the relevant literature to present depressive symptomatology [17,20]. Further investigation into these hypotheses is necessary.

With regard to PD patients, it is worth noting that patients on long term treatment indicated a higher preference for the dimension of *chance *on health locus of control (Table 3). It may be argued that as time of treatment increases, PD patients seem to change their illness beliefs and rely less on human and professional aspects regarding their medical care, instead turning to beliefs of power stemming from unpredictable external factors, like *chance*. This finding may be interpreted as the patients wishing to distance themselves from medical carers. If this is the case, it should be addressed appropriately by the health professionals involved in the peritoneal dialysis treatment of the participating dialysis units.

In addition, based on the means of the groups seen in the ANOVA (Table 3), PD patients in their initial stages of treatment seem to rely more on their *doctors *than the patients in the other categories. This finding may reflect the need of PD patients to rely more on the help of their medical helpers for undertaking the required training in peritoneal dialysis procedures as they are entering PD treatment.

In general, HD patients appear to endorse a more stable pattern of illness beliefs over time, as these patients, both those recently commenced and those on long term treatment appear to hold a similar weighting in the dimensions of health locus of control.

It is worth noting that findings in the area of illness beliefs of control are very limited. The results of one study reported in the literature, indicated no differences between HD and PD patients' health locus of control [41]. This psychological dimension has been well examined in HD patients in relation to adherence [42]. It is necessary however to take into consideration that methodological issues may account for differential findings, one of which is the possible cultural differences between the patient samples. It is suggested that examining cross-cultural differences regarding heath beliefs and attitudes in ESRD patients may provide answers to some of these queries.

This study has demonstrated that different QoL deficits, mental health problems and illness beliefs may characterize PD and HD patients. The findings of the present study could be used in the development of health care services and in-patient management. The role of health beliefs in particular may play an important role in the course of illness and treatment outcomes and could therefore be identified as a new area for psychological intervention in people with End Stage Renal Disease [43,44].

## Limitations

Limitations of the study may include the lack of investigating the effect of clinical factors such as adequacy of dialysis, hemoglobin level or other clinical parameters on the patients' perceptions of quality of life and mental health. The focus of the study however, was on QoL differences and adverse symptoms in HD and PD patients who recently commenced and those on long-term treatment.

## Conclusion

The main focus of the present study was to examine QoL in HD and PD patients and investigate differences between patients who recently commenced and those on long-term treatment. The results of the study provide evidence that the HD on long term of treatment had increased deficits in *physical, social *and *environmental *QoL, as well as in mental health.

Further, patients who had similar length of current treatment in the two treatment modalities, differ only with regards to their *environmental *well-being. Thus, in relation to differences between patients in early and later years of treatment, it appears that QoL deficits in HD patients become more extended over time, and seem to be more precisely signified by the factors in the *environmental *QoL domain. It may be argued that HD patients on long term dialysis appear to be more seriously compromised in their quality of life and mental health.

## Abbreviations

CAPD: Continuous Ambulatory Peritoneal Dialysis; ESRD: End Stage Renal Disease; HD: Haemodialysis; GHQ: General Health Questionnaire; MHLC: Multidimensional Health Locus of Control; PD: Peritoneal Dialysis; QoL: Quality of Life; WHOQOL-BREF: World Health Organisation quality of life instrument.

## Competing interests

The authors declare that they have no competing interests.

## Authors' contributions

All authors have read and approved the final manuscript. MG-C: Design of study, collection of data, analysis and write-up. PT: Collection of data, analysis and first draft. CSy: Collection of data, comments on draft. VT: Changes on the final version of the manuscript, additional points included in the discussion. CSo: Comments on final draft.

## Pre-publication history

The pre-publication history for this paper can be accessed here:


